# Brassinosteroid signaling may regulate the germination of axillary buds in ratoon rice

**DOI:** 10.1186/s12870-020-2277-x

**Published:** 2020-02-14

**Authors:** Huibin Xu, Ling Lian, Fuxiang Wang, Jiahuan Jiang, Qiang Lin, Hongguang Xie, Xi Luo, Yongsheng Zhu, Chuanying Zhuo, Jinlan Wang, Huaan Xie, Zhaowei Jiang, Jianfu Zhang

**Affiliations:** 1 Rice Research Institute, Fujian Academy of Agricultural Sciences, Fuzhou, 350019 Fujian China; 2 Key Laboratory of Germplasm Innovation and Molecular Breeding of Hybrid Rice for South China, Ministry of Agriculture and Rural Affairs, Fuzhou, 350003 Fujian China; 3 Incubator of National Key Laboratory of Germplasm Innovation and Molecular Breeding between Fujian and Ministry of Sciences and Technology, Fuzhou, 350003 Fujian China; 4 Fuzhou Branch, National Rice Improvement Center of China, P.R. China, Fuzhou, 350003 Fujian China; 5 Fujian Engineering Laboratory of Crop Molecular Breeding, P.R. China, Fuzhou, 350003 Fujian China; 6 Fujian Key Laboratory of Rice Molecular Breeding, P.R. China, Fuzhou, 350003 Fujian China; 7 Base of South China, State Key Laboratory of Hybrid Rice, P.R. China, Fuzhou, 350003 Fujian China; 8 Bureau of Agricultural and Rural Affairs of Youxi County, Sanming, 350108 Fujian China

## Abstract

**Background:**

Rice ratooning has traditionally been an important component of the rice cropping system in China. However, compared with the rice of the first harvest, few studies on factors effecting ratoon rice yield have been conducted. Because ratoon rice is a one-season rice cultivated using axillary buds that germinate on rice stakes and generate panicles after the first crop’s harvest, its production is mainly affected by the growth of axillary buds. The objectives of this study were to evaluate the sprouting mechanism of axillary buds to improve the ratoon rice yield.

**Results:**

First, we observed the differentiation and growth dynamics of axillary buds at different nodes of Shanyou 63, and found that they differentiated from bottom to top before the heading of the mother stem, and that they developed very slowly. After heading they differentiated from top to bottom, and the ones on the top, especially the top 2nd node, developed much faster than those at the other nodes. The average length and dry weight of the axillary buds were significantly greater than those at other nodes by the yellow ripe stage, and they differentiated into pistils and stamens by 6 d after the yellow ripe stage. The morphology of vegetative organs from regenerated tillers of Shanyou 63 also suggested the superior growth of the upper buds, which was regulated by hormones, in ratoon rice. Furthermore, a comprehensive proteome map of the rice axillary buds at the top 2nd node before and after the yellow ripe stage was established, and some proteins involved in steroid biosynthesis were significantly increased. Of these, four took part in brassinosteroid (BR) biosynthesis. Thus, BR signaling may play a role in the germination of axillary buds of ratoon rice.

**Conclusions:**

The data provide insights into the molecular mechanisms underlying BR signaling, and may allow researchers to explore further the biological functions of endogenous BRs in the germination of axillary buds of ratoon rice.

## Background

Rice (*Oryza sativa* L.) represents an important food crop worldwide and can produce panicle-bearing secondary tillers (i.e., ratoons) following harvest [1]. The production of a second rice crop in one cropping season is known as ratooning. The ratoon crop develops by regenerating rice tillers from nodal buds of the stubble that is left behind after the first seasonal rice harvest [2, 3]. In areas where adequate water is available after the main season, rice ratooning can be practiced as an alternative to double cropping [4]. It requires fewer inputs, such as fertilizers, than the main crop and can often provide a net profit for the grower when the main crop barely recovers input costs [5]. In China, ratoon rice dates back 1700 years [6, 7]. In the 1930s to 1940s, studies of the morphological development of ratooning tillers began, and a number of ratoon rice varieties were bred in China in the 1960s and 1970s [8]. Since the 1980s, with the breeding of a number of new hybrid rice varieties having high regeneration capacities, ratooning rice has become a new cropping system promoted in large areas of southern China. In 1997, the planting area of ratoon rice expanded to 7.5 × 10^5^ hm^2^ in China [6, 7]. From 1998 to 2002, super high-yielding demonstration area of ratoon rice was established in Youxi County, Sanming City, Fujian Province, China, and its accumulated planting area covered 1097.73 hm^2^ in 5 years. The average yield was 10,664 kg/hm^2^ in the first season and 6537 kg/hm^2^ in the ratooning season, leading to a total annual yield of 17,201 kg/hm^2^ [9]. There are 1.07 × 10^7^ hm^2^ single cropping paddy fields in southern China, with 3.3 × 10^6^ hm^2^ being suitable for planting ratoon rice. With a ratoon rice yield of 17,201 kg/hm^2^, this area could produce 5.68 × 10^10^ kg of rice per year. This is of great significance for grain production and its strategic development in China, which feeds 22% of the world’s population with 7% of the world’s cultivated land.

Ratooning rice is the rice tillers which regenerate from the nodal buds of stubble [10]. The new tillers are regenerated from the first or main crop that is harvested in first season [3]. The physiological parameters are different between main and ratoon crop. For instance, panicle development and heading of ratoon rice is less than that of main rice. Low yield of ratoon crop usually depends on the reduction in number of productive tillers and short growth duration [10]. In the main rice crop, sugars and starches accumulated in leaves and culm are translocated to grain after flowering. The hypothesis is that carbohydrates accumulate in the culms again near main crop maturity, which may make preparations for ratoon growth and development. Therefore, effective application of ratoon-specific agronomic measures may provide a great potential for yield of ratooning rice [3].

The factors affecting the growth and development of ratoon rice include the varieties developed, water and fertilizer management, stubble height, plant protective practices, and external environmental factors, such as temperature and light, which affect the yield of ratoon rice [10]. Among these factors the most important is varietal development. Previous research focused on the effects of external factors, such as planting date, water and fertilizer management, and other environmental factors, on ratoon crop production. Although the varietal and stubble height effects on the yield of ratoon rice have been studied, their potentials and their impacts were limited [3, 5, 10]. Therefore, the most effective way to improve the yield of ratoon rice is by fully understanding the sprouting mechanism of axillary buds and promoting the ratooning capacity. The germination of axillary buds is very important for the stable and high yielding cultivation of ratoon rice, but its mechanism remains unknown [7]. Consequently, further studies are required to decipher the sprouting mechanism of axillary buds to optimize cultivation techniques for bud and tiller promotion in ratoon rice.

The germination of axillary buds is influenced by apical dominance [11], which is regulated by hormones [12]. Brassinosteroids (BRs) are a class of plant-specific steroid hormones characterized by polyhydroxylated sterol structures that have significant growth-promoting activities, which are essential for normal plant growth and development [13, 14]. They participate in the regulation of numerous vital physiological processes in plants, such as elongation, germination, photomorphogenesis, immunity, and reproductive organ development [15–19]. One of the most active BRs can prominently increase coleoptile length and decrease root length in rice [20]. BRs have particularly strong growth-promoting effects in stems of seedlings and young plants, mainly by promoting the expression of genes involved in cell elongation and wall extensibility [21, 22]. Although the recent isolation and functional characterization of BR-related genes have greatly expanded our knowledge, little is known regarding BR signaling in the development of rice axillary buds. Because plants utilize complicated mechanisms to maintain BR homeostasis in vivo, more research is needed to enable the genetic modification of rice axillary bud development through the fine-tuning of BR biosynthesis and signaling pathways. Consequently, we established a comprehensive proteome map of the rice axillary bud before and after the yellow ripe stage to determine the proteins involved in BR signaling. Furthermore, we investigated the possible roles of BRs in the germination of axillary buds in ratoon rice.

The sprouting of axillary buds is a complex process during which a series of physiological and biochemical reactions occur, including signal transduction, reactivation of metabolism and redox homeostasis regulation. These biological processes are catalyzed and regulated by various proteins, so it is worthy to construct a comparative proteome profile for buds during its sprouting. Moreover, the role of individual protein could be evaluated by integrating proteomic data into a network of regulatory pathways [23]. Recently, two-dimensional gel electrophoresis and multidimensional protein identification technology were commonly methods to recognize and quantify proteins, which were not suitable for all types of proteins including low-abundance proteins. It only obtained proteins under special conditions of interest or in a specific organ. However, the combination of peptide labeling with liquid chromatography-tandem mass spectrometry (LC-MS/MS) has given new insights into dynamical and functional aspects of proteins in plant [24]. In this study, we detailly observed the growth law of axillary buds in Shanyou 63 (cross of Minghui 63 and Zhenshan 97B). Subsequently, we constructed protein expression profile of rationing bud in Minghui 63 (strong ratooning ability), Zhenshan 97B (weak ratooning ability) and Shanyou 63 by isobaric tags for relative and absolute quantification (iTRAQ). Our work obtained the comprehensive information on rationing bud proteome and identified differentially expressed proteins involved in its sprouting. Meanwhile, we identified the metabolic pathways that may play important role in bud sprouting. This will provide more insight into understanding of the sprouting mechanism of rice axillary buds.

## Results

### The differentiation of axillary buds

**Fig. 1:**
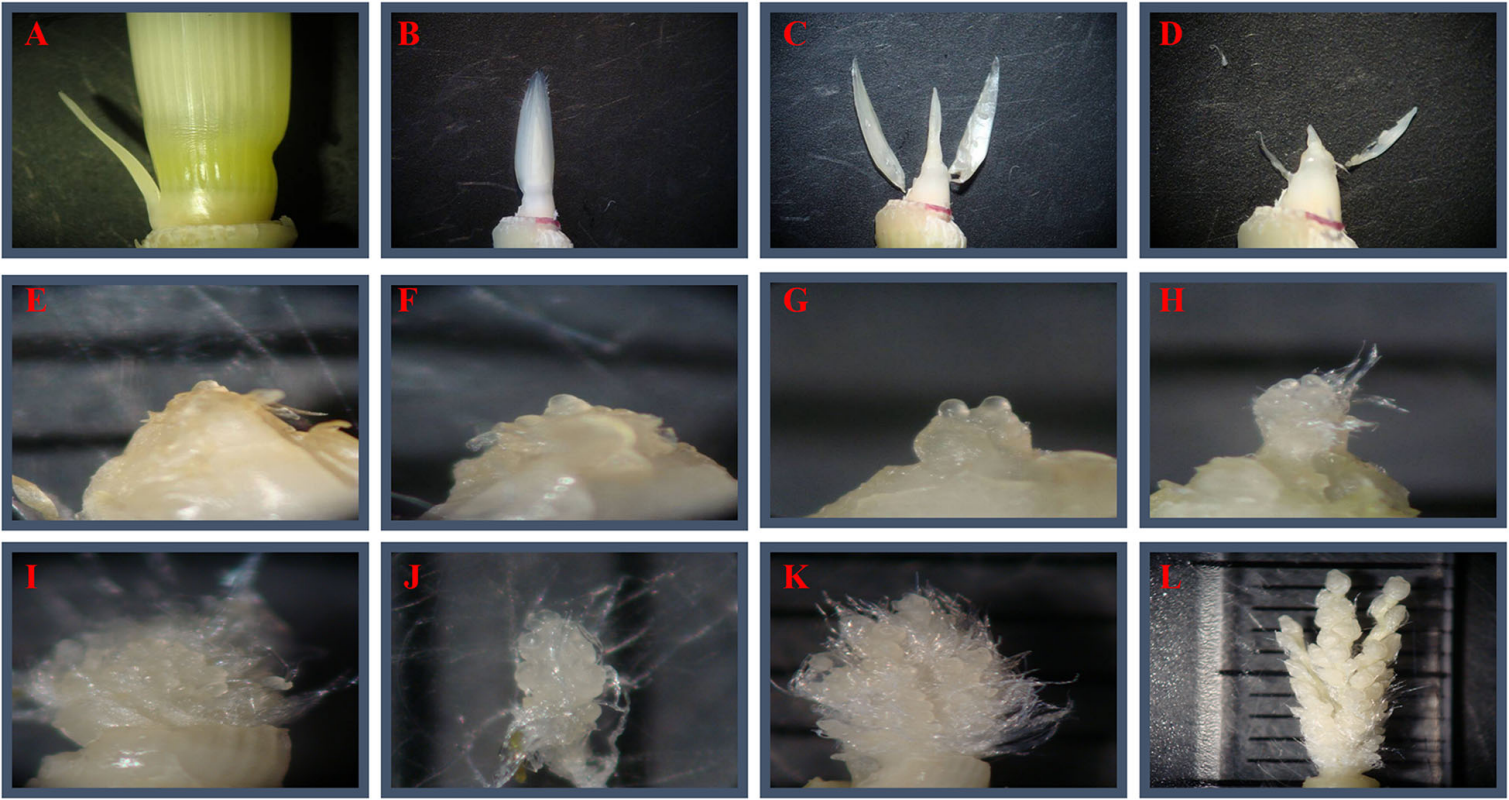
The microscopic observations of the differentiation processes of axillary buds. (**a**) Axillary buds during the bract differentiation stage. (**b**) Bract differentiation stage (The prophyll is 6–10 mm long). (**c**) Bract differentiation stage (The first leaf is 4–7 mm long). (**d**) Bract differentiation stage (The second leaf is 1–2 mm long). (**e**) Bract differentiation stage (the third leaf is 0.2–0.5 mm long). (**f**) Bract differentiation stage (The first bract is 0.1–0.2 mm long). (**g**) Primary branch differentiation (0.1–0.5 mm, the prophyll is 20–30 mm long). (**h**) Secondary branch differentiation (0.5–1.2 mm, the prophyll is 40–60 mm long). (**i**) Spikelet differentiation (1.0–2.5 mm). (**j**) Closeup of spikelet differentiation. (**k**) The initial stage of pistil and stamen differentiation (3–5 mm). (**l**) The late stage of pistil and stamen differentiation (10 mm)

**Fig. 2:**
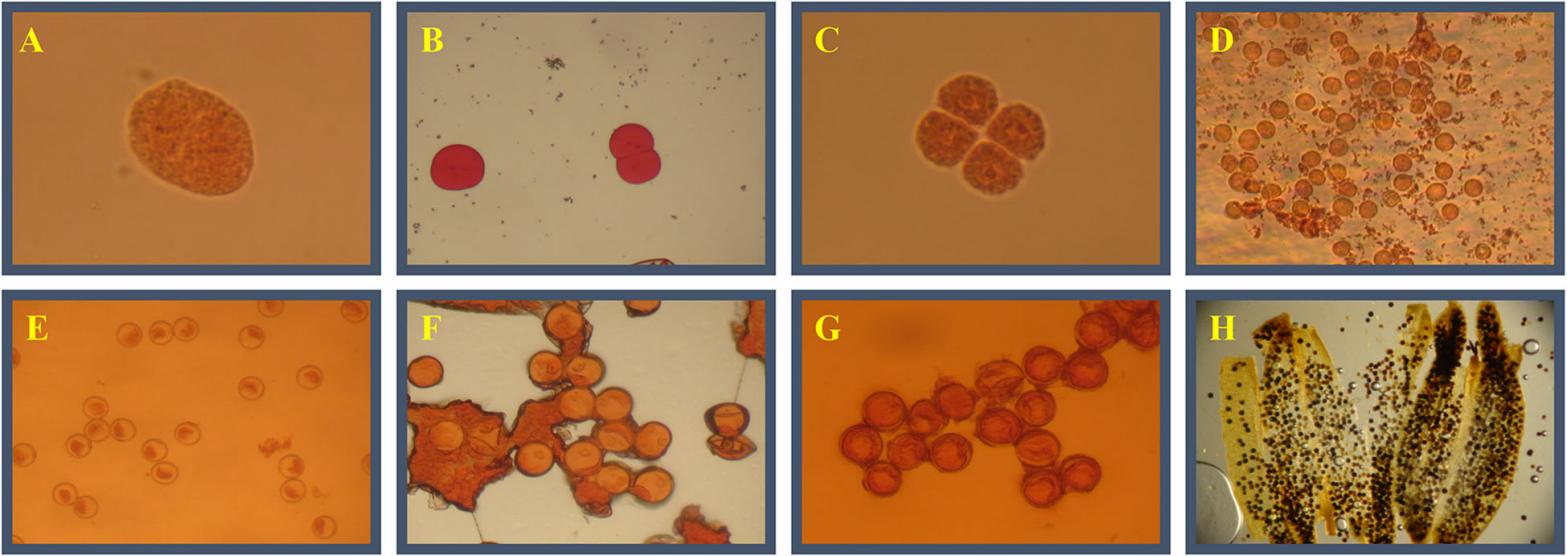
Young spike differentiation of axillary buds. (**a**) Pollen mother cells (the pulvinus distance is − 3- (− 5) cm, glume length is 50%). (**b**) Dyad (the pulvinus distance is − 4-(+ 2) cm, panicle length is 7–10 cm, glume length is 55–75%). (**c**) Tetrad. (**d**) The initial microspore stage (The pulvinus distance is − 4-(+ 2) cm, glume length is 60–80%). (**e**) Mononuclear centralization of the microspore (the pulvinus distance is 4–8 cm, glume length is 70–80%). (**f**) Single nucleus to the side (the pulvinus distance is 8–15 cm, glume length is 85–95%, blade internode is 0.5–1 cm). (**g**) Nuclear division (the pulvinus distance is 12–20 cm, glume length is 100%). (**h**) Pollen maturity (the panicle occipital interval is greater than − 2 cm, the spike neck length is 5 cm)

Ratoon rice has two crop harvests. The first crop is harvested at the full heading stage and the second crop is harvested at the yellow ripe stage. In general, there are five stem leaves and six internodes in the above-ground parts of the late-maturing hybrid rice varieties cultivated for ratoon rice. Except for the top 1st node, the axillary buds at the other nodes can develop from the leaf axil and germinate to form a regenerative tiller under suitable conditions. A microscopic examination of the axillary buds of Shanyou 63 showed that until 12 d before the yellow ripe stage in the first season, the axillary buds remained at the bract differentiation stage (Fig. 1a-f, Table 1). From 9 d before the yellow ripe stage, the differentiation of axillary buds began the new stage of young panicle differentiation from top to bottom (Fig. 1g, Table 1). The axillary buds at the top 2nd and 3rd nodes differentiated into primary branches at 9 d before the yellow ripe stage (Fig. 1g, Table 1), into secondary branches at 3 d before the yellow ripe stage (Fig. 1h, Table 1), into spikelets at 3 d after the yellow ripe stage (Fig. 1i, j, Table 1), and some axillary buds at the top 2nd node differentiated into pistils and stamens at 6 d after the yellow ripe stage (Fig. 1k, l, Table 1). The panicle differentiation of axillary buds at the top 4th and 5th nodes lagged. The axillary buds at the top 4th node only entered into primary branch differentiation during the yellow ripe stage in the first season and the secondary branch differentiation at 6 d after the yellow ripe stage. Only some of the axillary buds at the top 5th node entered into primary branch differentiation during the yellow ripe stage and into the secondary branch differentiation at 6 d after the yellow ripe stage (Table 1). Observations of the axillary buds at different nodes revealed that they began to differentiate from bottom to top before the heading of the mother stem, but they developed very slowly. After the heading of the mother stem, they differentiated from top to bottom, and the axillary buds on the top developed much faster than the other buds (Fig. 2).Thus, upper bud growth was superior in ratoon rice.

**Table 1:** The differentiation process of axillary buds at different nodes

Axillary buds at different nodes	12d before yellow ripe stage	9d before yellow ripe stage	6d before yellow ripe stage	3d before yellow ripe stage	The yellow ripe stage	3d after yellow ripe stage	6d after yellow ripe stage
The top 2nd	I	II	II	III	III~IV	IV	IV~V
The top 3rd	I	II	II	III	III	III~IV	IV
The top 4th	I	I	I	I	II	II	III
The top 5th	I	I	I	I	I~II	II	II~III
The top 6th	I	I	I	I	I	I~II	II

I. Bract differentiation stage, II. Primary branch differentiation stage, III. Secondary branch differentiation stage, IV. Spikelet differentiation stage, V. Pistil and stamen differentiation stage

### The growth dynamics of axillary buds

Based on observations of Shanyou 63, there was a long latency period for axillary buds on the stem, growing only 2 mm before the heading of the mother stem. This was owing to the control of apical dominance. From 12 to 6 d before the yellow ripe stage, their lengths were only 6.2–12.6 mm, and at 3 d before the yellow ripe stage, most axillary buds at the top 2nd and 3rd nodes began to elongate, as did some axillary buds at the top 4th and 5th nodes (Table 2). At the yellow ripe stage, the average lengths and dry weights of axillary buds at the top 2nd node were 80.1 mm and 55.67 mg, respectively, those of the top 3rd node were 68.4 mm and 48.23 mg, respectively, and those of the top 4th and 5th nodes were 34.8–4.63 mm and 16.68–18.07 mg, respectively (Tables 2, 3). By comparing the lengths and dry weights of axillary buds at different nodes, we found that they did not show significant differences before the yellow ripe stage, and they all grew slowly. However, at 3 d before the yellow ripe stage, all the axillary buds started to grow significantly and the growth trends of the top 2nd and 3rd were the greatest (Tables 2, 3).

**Table 2:** The length of axillary buds at different nodes during ratooning season

Axillary buds at different nodes(cm)	12d before yellow ripe stage	9d before yellow ripe stage	6d before yellow ripe stage	3d before yellow ripe stage	The yellow ripe stage	3d after yellow ripe stage
The top 2nd	0.62 ± 0.005	0.74 ± 0.000*	0.78 ± 0.003*	6.43 ± 0.001***	8.01 ± 0.001***	10.40 ± 0.692***
The top 3rd	1.21 ± 0.001^###^	1.23 ± 0.001^###^	1.26 ± 0.001^###^	5.84 ± 0.008***^###^	6.84 ± 0.013***^###^	8.00 ± 0.012***^##^
The top 4th	0.94 ± 0.002^###^	1.13 ± 0.000**^###^	1.17 ± 0.001**^###^	2.02 ± 0.017***^###^	4.63 ± 0.001***^###^	5.30 ± 0.005***^###^
The top 5th	0.72 ± 0.000	0.74 ± 0.000	0.78 ± 0.004	3.53 ± 0.019***^###^	3.48 ± 0.005***^###^	4.20 ± 0.013***^###^
The top 6th	0.63 ± 0.001	0.64 ± 0.002^#^	0.69 ± 0.003	2.04 ± 0.011***^###^	2.84 ± 0.005***^###^	3.80 ± 0.023***^###^

The length of axillary buds in different time periods and different nodes was taken as the index for statistical analysis. *, comparison of axillary bud length between different time periods and 12d before yellow ripe stage, ^*^p < 0.05, ^**^p < 0.01,^***^p < 0.001. #, comparison of axillary bud length between different nodes and the top 2nd, ^#^*p* < 0.05, ^##^*p* < 0.01, ^###^*p* < 0.001. All values were shown as the means ± standard error of the mean of three replicates

**Table 3:** The dry weight of axillary buds at different nodes during ratooning season

Axillary buds at different nodes (mg)	12d before yellow ripe stage	9d before yellow ripe stage	6d before yellow ripe stage	3d before yellow ripe stage	The yellow ripe stage	3d after yellow ripe stage
The top 2nd	4.07 ± 0.004	4.24 ± 0.002^*^	4.57 ± 0.028^**^	33.16 ± 0.490^***^	55.67 ± 9.217^***^	67.03 ± 5.849^***^
The top 3rd	6.16 ± 0.004^###^	7.02 ± 0.020^***###^	6.85 ± 0.172^*###^	29.53 ± 2.821^***#^	48.23 ± 1.200^***#^	58.38 ± 6.043^***#^
The top 4th	5.27 ± 0.006^###^	5.67 ± 0.005^**###^	5.84 ± 0.027^**###^	6.79 ± 0.027^***###^	18.07 ± 0.280^***###^	29.27 ± 0.488^***###^
The top 5th	4.47 ± 0.009^##^	4.76 ± 0.011^*##^	4.93 ± 0.075^*^	13.48 ± 1.330^***###^	16.68 ± 0.778^***###^	19.47 ± 1.658^***###^
The top 6th	4.68 ± 0.008^###^	4.77 ± 0.015^##^	4.89 ± 0.009^*#^	6.87 ± 0.074^***###^	10.55 ± 0.609^***###^	14.83 ± 4.295^**###^

The dry weight of axillary buds in different time periods and different nodes was taken as the index for statistical analysis. *, comparison of axillary bud dry weight between different time periods and 12d before yellow ripe stage, ^*^p < 0.05, ^**^p < 0.01,^***^p < 0.001. #, comparison of axillary bud dry weight between different nodes and the top 2nd, ^#^p < 0.05, ^##^p < 0.01, ^###^p < 0.001. All values were shown as the means ± standard error of the mean of three replicates

### Morphological developments of regenerated tillers

Observations of the morphology of vegetative organs regenerated from tillers revealed that the total number of leaves at the top 2nd and 3rd nodes was usually three, and that of the top 4th and 5th nodes was usually four. The lengths of organs at each node were measured (Table 4). Most of the first leaves were atrophic, with lengths of only 1–3 cm, and were still enclosed in the sheathes of prophylls for a long time, which made it difficult to determine the leaf age during the early stage of young spike development (Table 4). Thus, the elongation dynamics of the vegetative organs were measured at the same time. Finally, the lengths and ages of prophylls were used as indicators to determine the developmental processes of young panicles in the 1st-5th stages. The pulvinus distance of the top two leaves, the distance of spicae and leaves, the spikelet length, and the length between the top two internodes were used as the indicators to determine the developmental processes of young panicles in the 6th–8th stages (Additional file 1: Figure S1, Table 5).

**Table 4:** The length of vegetative organs in nodes of regenerated tillers

Organ	Length of organs (three leaves at each node, cm)					Length of organs (four leaves at each node, cm)					
	P	1	2	3	N	P	1	2	3	4	N
Leaf	–	2.8	22.9	24.1	–	–	2.8	14.4	25.3	21.1	–
Leaf sheath	6.4	12.7	15.4	20.7	–	3.4	8.8	12.8	15.1	20.8	–
Internode	0.4	1.8	5.6	15.3	22.1	0.3	0.7	2.7	7.1	14.5	21.4

*P* prophyll, *N* neck spike

**Table 5:** Diagnostic indicators for the developmental morphology of young panicles in regenerative tillers

Developmental Stage	Dynamics of organ elongation	The length and age of leaf		Other diagnostic indicators
		Tillers with three leaves	Tillers with four leaves	
①Initial stage of bract differentiation	Initial elongation of the top 3rd leaf	0.6~1 cm (prophyll)	4~5 cm (prophyll)	
②Initial stage of primary branch differentiation	Obvious elongation of the top 3rd leaf	2~3 cm (prophyll)	one mature leaf and one new leaf (1/10 of the mature leaf)	
③Initial stage of secondary branch differentiation	Initial elongation of the top 2nd leaf	4~5 cm (prophyll)	one mature leaf and one new leaf (5/10 of the mature leaf)	
④Initial stage of spikelet differentiation	Obvious elongation of the top 2nd leaf	one mature leaf and one new leaf (1/10 of the mature leaf)	two mature leaves and one new leaf (1/10 of the mature leaf)	
⑤Initial stage of pistil and stamen differentiation	Initial elongation of the top 1st leaf	one mature leaf and one new leaf (5/10 of the mature leaf)	two mature leaves and one new leaf (5/10 of the mature leaf)	
⑥Pollen mother cell and meiosis stage	Obvious elongation of the top 1st leaf sheath	two mature leaves and one new leaf (7/10 of the mature leaf)	three mature leaves and one new leaf (7/10 of the mature leaf)	The pulvinus distance of the top two leaves is -5 cm, spikelet length is 50%.
⑦Initial stage of pollen filling	Initial elongation of the top 1st internode	three mature leaves	four mature leaves	The pulvinus distance of the top two leaves is 0 cm, spikelet length is 70%.
⑧Initial stage of pollen maturity	Initial elongation of panicle neck internode			The panicle occipital interval is above-2 cm, the spike neck length is 5 cm

### Proteomic analysis of the growth dynamics of axillary buds

**Fig. 3:**
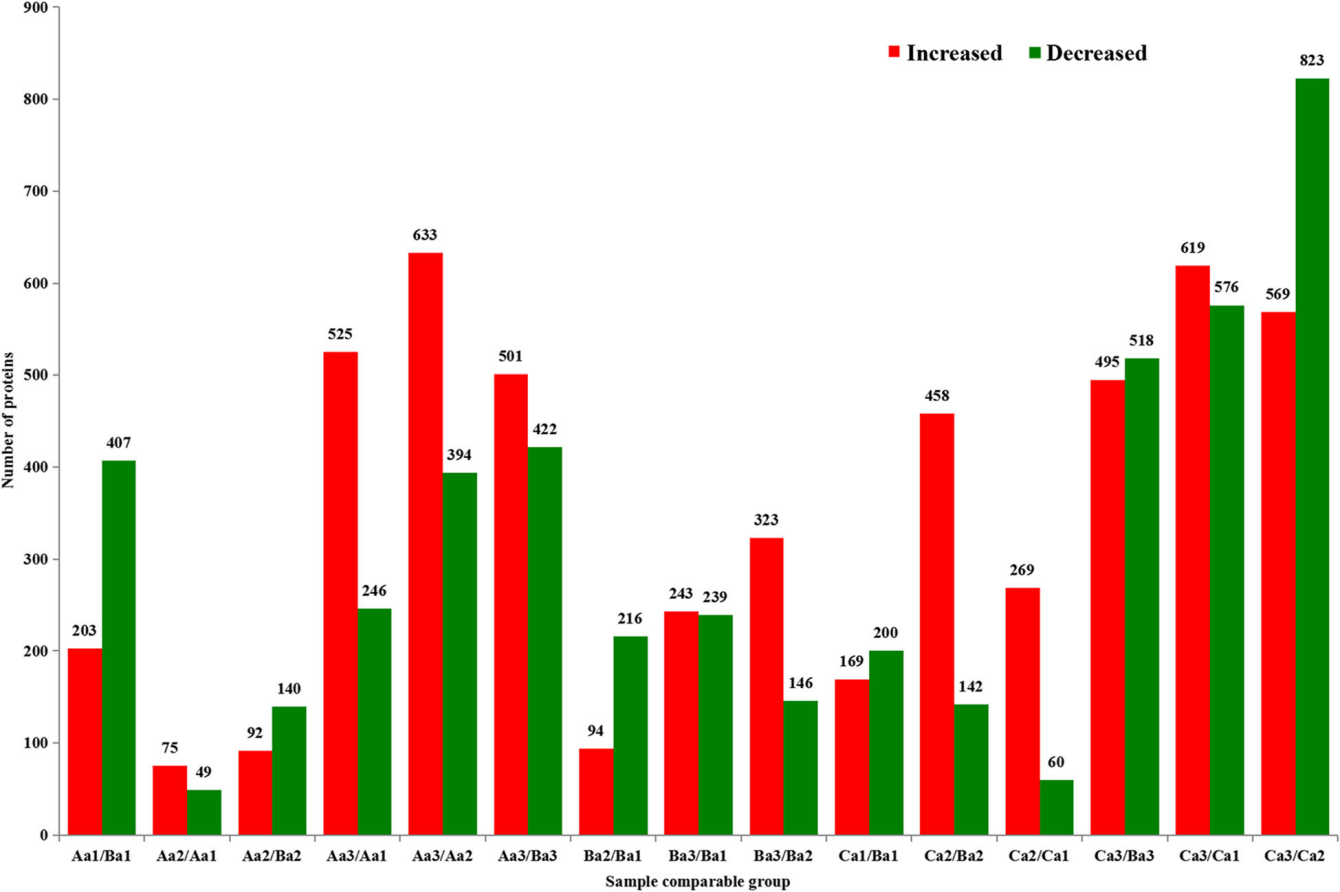
Differentially abundant proteins in rice axillary buds at the top 2nd node. Aa1: Minghui 63, 3 d before the yellow ripe stage; Aa2: Minghui 63, yellow ripe stage; Aa3: Minghui 63, 3 d after the yellow ripe stage; Ba1: Zhenshan 97B, 3 d before the yellow ripe stage,; Ba2: Zhenshan 97B, yellow ripe stage; Ba3: Zhenshan 97B, 3 d after the yellow ripe stage; Ca1: Shanyou 63, 3 d before the yellow ripe stage; Ca2: Shanyou 63, yellow ripe stage; Ca3: Shanyou 63, 3 d after the yellow ripe stage

The germination rates of axillary buds in different rice varieties with different ratooning capabilities are different, and the germination rates of axillary buds at different nodes of the same rice variety are also different. Among them, axillary buds at the top 2nd node of high ratooning varieties had the greatest rates. According to our previous research, all the axillary buds grew significantly at the yellow-ripe stage (Tables 2, 3). To combine our large-scale axillary bud proteome analysis with quantitative information on proteins modulated during the yellow ripe stage, we used the Tandem Mass Tag (TMT) method [25]. Protein extracts from rice axillary buds at the top 2nd nodes of Minghui 63, Zhenshan 97B, and Shanyou 63 were analyzed by the combination of one-dimensional gel electrophoresis (1-DE) with LC-MS/MS. About 100 μg protein was load in 1-DE and visualized slices were digested by trypsin. Then all proteins were identified using LC-MS/MS analysis. The proteins with fold changes (FC) greater than 1.3 and *p*-values less than 0.05 were identified as differentially abundant proteins. In this study, 9846 differentially abundant proteins were identified, of which 5268 (53.5%) were increased and 4578 (46.5%) were decreased (Fig. 3, Additional file 1: Table S1). There were much more differentially abundant proteins at 3 d after the yellow ripe stage, suggesting that many genes or proteins were stimulated and expressed when the axillary buds germinated (Fig. 3, Additional file 1: Table S2). At the same time, by comparing the differentially abundant proteins of different combinations, it was found that the strongly regenerative variety Shanyou 63 and weakly regenerative variety Zhenshan 97B had the greatest numbers of differentially abundant proteins at 3 d after the yellow ripe stage. Therefore, we focused on an analysis of these differentially abundant proteins and screened for proteins involved in the germination of axillary buds.

### Gene ontology (GO) annotation and subcellular classify of the differentially abundant proteins

**Fig. 4:**
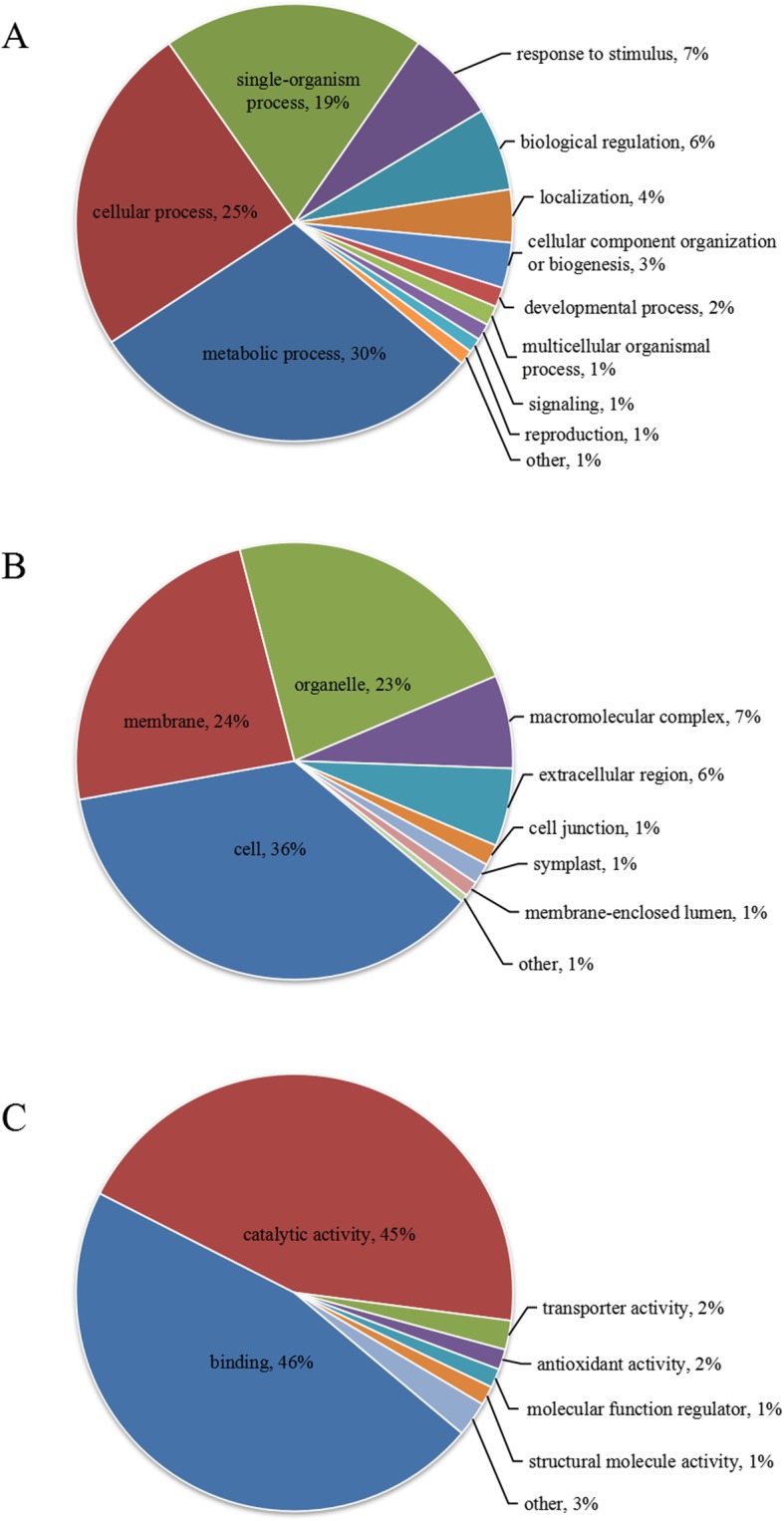
Gene ontology (GO) annotation of the various proteins. (**a**) Pie charts of over-represented terms in the biological process ontology. (**b**) Pie charts of over-represented terms in the cellular component ontology. (**c**) Pie charts of over-represented terms in the molecular function ontology. Different colors represent different GO terms

Proteins were classified by GO annotation into three categories: biological process, cellular compartment, and molecular function. Based on biological process ontology, the proteins were classified into functional categories, including metabolic process (30%), cellular process (25%), single-organism process (19%), response to stimulus (7%), biological regulation (6%), localization (4%), cellular component organization or biogenesis (3%), developmental process (2%), multicellular organismal process (1%), signaling (1%), reproduction (1%), and other (1%) (Fig. 4a). Based on cellular component ontology, the proteins were classified into functional categories, including cell (36%), membrane (24%), organelle (23%), macromolecular complex (7%), extracellular region (6%), cell junction (1%), symplast (1%), membrane-enclosed lumen (1%), and other (1%) (Fig. 4b). Based on molecular function ontology, the proteins were classified into functional categories, including binding (46%), catalytic activity (45%), transporter activity (2%), antioxidant activity (2%), molecular function regulator (1%), structural molecule activity (1%), and other (3%) (Fig. 4c). The GO annotation revealed that the proteins were located primarily in cells, membranes, and organelles, played roles in metabolic processes, and had binding and catalytic activities (Fig. 4).

The cells of eukaryotic organisms are elaborately subdivided into functionally distinct membrane-bound compartments. Consequently, we also used the Wolfsport software to predict the subcellular structures of the differentially abundant proteins (Additional file 1: Figure S2), which included chloroplast (34%), cytoplasm (24%), nucleus (21%), extracellular (7%), plasma membrane (6%), mitochondria (4%), vacuolar membrane (2%), cytoskeleton (1%), and endoplasmic reticulum (1%) (Additional file 1: Figure S2). These results reflected the gradual increase in metabolic activity that occurs in rice axillary buds during germination.

### BRs may mediate the germination of axillary buds

**Fig. 5:**
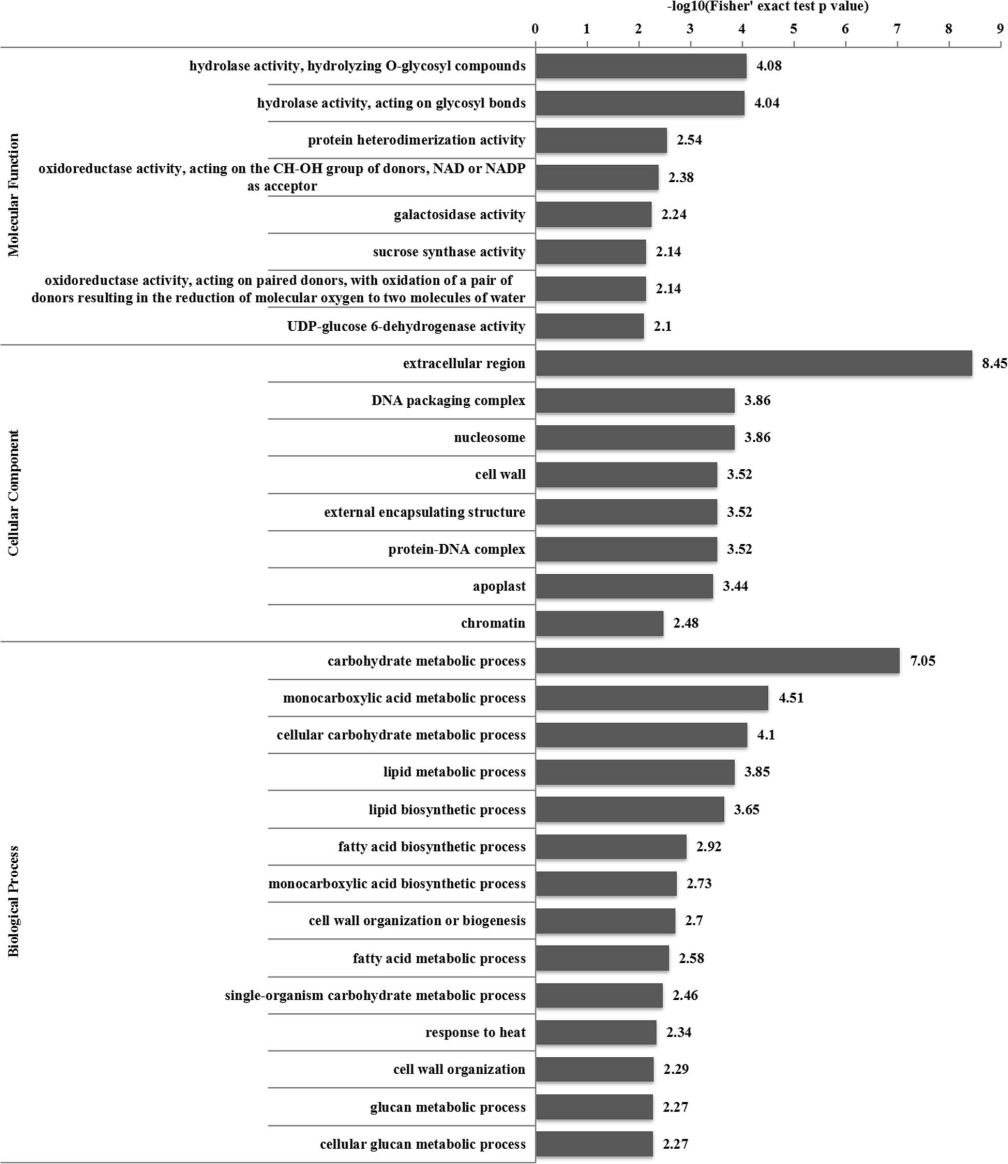
GO enrichment analysis. *p*-value < 0.05

**Fig. 6:**
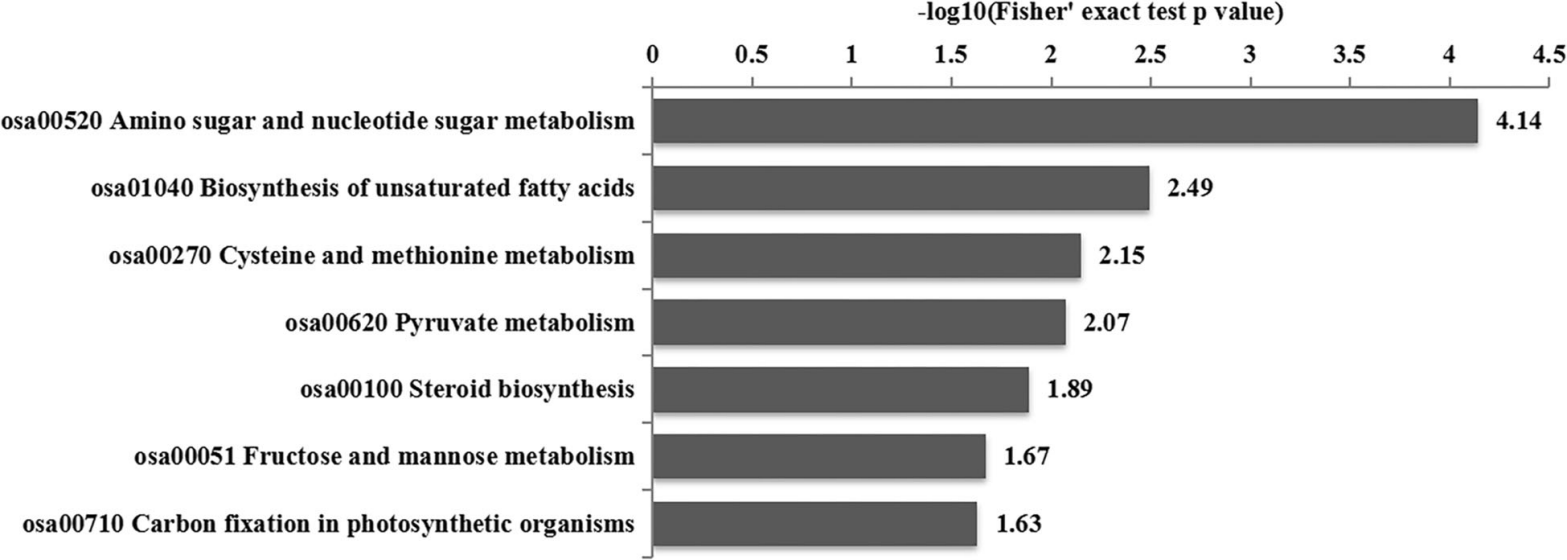
KEGG pathway enrichment analysis. p-value < 0.05

To determine whether differentially abundant proteins had significant enrichment trends in particular functional types, GO, Kyoto Encyclopedia of Genes and Genomes (KEGG) and domain enrichment analyses were performed (Figs. 5, 6; Additional file 1: Figure S3). The most abundant proteins were those with hydrolase, protein heterodimerization, oxidoreductase, galactosidase, sucrose synthase and UDP-glucose 6-dehydrogenase activities, which are involved in carbohydrate, monocarboxylic acid, cellular carbohydrate, and lipid metabolic processes, as well as lipid and fatty acid biosynthetic processes (Fig. 5). Based on the results of the KEGG enrichment analysis, the detected enzymes were involved in amino sugar and nucleotide sugar metabolism, unsaturated fatty acid biosynthesis, cysteine and methionine metabolism, pyruvate metabolism, steroid biosynthesis, fructose and mannose metabolism, and carbon fixation in photosynthetic organisms (Fig. 6).

During the germination of axillary buds, a series of physiological and biochemical reactions occur, and metabolic processes are necessary. In addition, germination is also regulated by hormones. There were differentially abundant proteins involved in steroid biosynthesis between Shanyou 63 and Zhenshan 97B (Additional file 1: Figure S4). BRs are plant steroidal hormones that regulate the growth and development of plants. In the steroid biosynthetic pathway, one branch is BR biosynthesis. Based on the KEGG enrichment analysis, there were four significantly increased BR biosynthesis-related proteins (Additional file 1: Figure S4). Thus, BR signaling may play a role in the germination of axillary buds in ratoon rice. The identification of these proteins will help to determine how BR signaling is involved in the germination of rice axillary buds. Thus, these proteins were selected for future studies.

### Protein expression as assessed by a real-time quantitative reverse transcription PCR (qRT-PCR) analysis

**Fig. 7:**
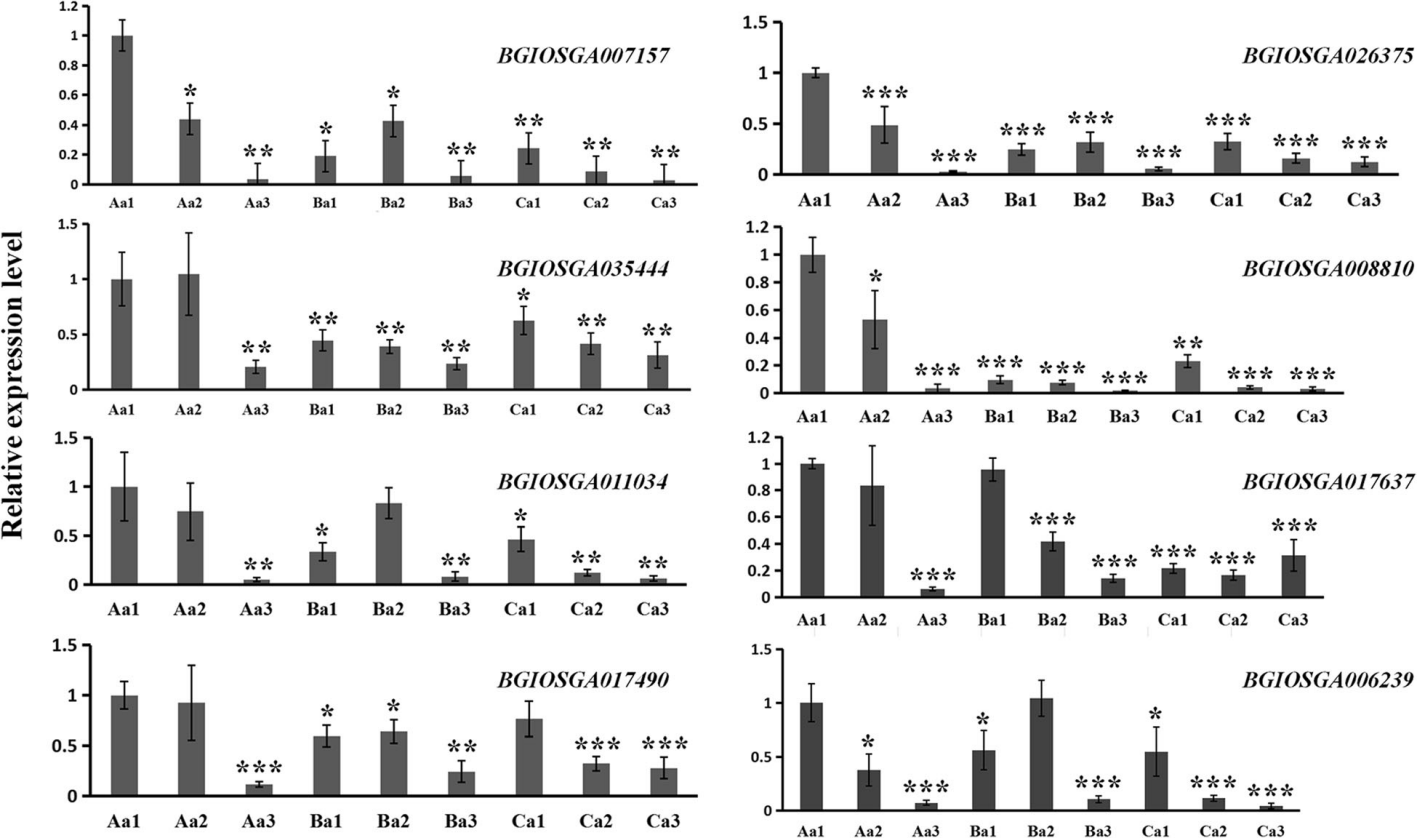
The expression levels of differentially abundant proteins in axillary buds before and after the yellow ripe stage as assessed by a qRT-PCR analysis. Values presented are the means ± SDs from three different duplicates (*n* = 3). Significant differences from 3 d before the yellow ripe stage of Minghui 63 are indicated by asterisks (Student’s *t*-test, ^*^P < 0.05, ^**^P < 0.01, ^***^P < 0.001)

To confirm the accuracy and reproducibility of the proteomics analysis results, eight of the differentially abundant proteins that regulated the steroid biosynthesis, fatty acid biosynthesis, and tyrosine metabolism (Additional file 1: Table S1) were selected for qRT-PCR verification. RNA samples from the axillary buds of Minghui 63, Zhenshan 97B, and Shanyou 63 were used as templates. The expression levels of the eight genes all decreased after the yellow ripe stage in three varieties, and they all expressed higher in strong ratooning variety than weak ratooning variety during the yellow ripe stage, except BGIOSGA006239 (glutathione S-transferase zeta 1) (Fig. 7). Among them, BGIOSGA007157 (cycloartenol synthase) took part in steroid biosynthesis, BGIOSGA035444 (long chain acyl-CoA synthetase 2) took part in fatty acid biosynthesis, BGIOSGA011034 (mannose-1-phosphate guanylyltransferase), BGIOSGA017490 (glucose-1-phosphate adenylyltransferase), BGIOSGA008810 (bifunctional dTDP-4-dehydrorhamnose 3,5-epimerase/dTDP-4-dehydrorhamnose reductase), and BGIOSGA026375 (UDP-glucuronic acid decarboxylase 2) took part in amino sugar and nucleotide sugar metabolism, BGIOSGA017637 (farnesyl pyrophosphate synthase 1) took part in terpenoid backbone biosynthesis, and BGIOSGA006239 (glutathione S-transferase zeta 1) took part in tyrosine metabolism. Thus, we identified some significantly abundant proteins that took part in steroid biosynthesis, like BGIOSGA007157((terpene cyclase)), and they may have roles in BR signaling during the regulation of axillary bud germination.

## Discussion

In the southern China, rice ratooning has traditionally been an important component of the rice cropping system. In this area, the production season is not long enough for a second rice planting, but rice ratooning provides an opportunity for a potential second harvest and also adds biomass back into the soil. The ratoon rice requires fewer inputs, such as fertilizers, than the main crop and can often provide a net profit for the grower when the main crop barely recovers input costs. However, compared with the first harvest, there are limited studies on factors that affect the yield of ratoon rice. Because ratoon rice is a one-season rice cultivated using axillary buds that germinate on rice stakes and generate panicles after the first crop’s harvest, its production can be affected by many factors [1–5]. Among them, the variety used is the most important factor. Varieties primarily determine the optimal yield potentials, while high tillering potential and tiller vigor are essential for maximizing the ratooning potential.

According to our previous research, the differentiation of the first three leaf primordia in axillary buds was closely related to the growth of mother stem. In the observation of several ratooning rice varieties, under the common cultivation conditions, the sprouting rate of the 2nd bud was 70–80%, the 3rd bud was 60–70%, the 4th bud was 20–40%, and the 5th bud was 10–15%. The number of grains per panicle was only one third of the mother stem, among which the 3rd was slightly larger, the 2nd and 4th were next, and the 5th was slightly smaller. The seed setting rate and 1000 grain weight decreased with the node down. Therefore, the 2nd and 3rd tillers made up about 80% of the total yield.

To improve the yield of the ratoon rice, we studied the differentiation and growth of the auxiliary buds at different nodes, the developmental traits of the pollen grains, and the morphology of the stem organs of Shanyou 63. The differentiation of the stem auxiliary buds began from the bottom to top before the heading of the mother stem, while after heading, the axillary buds on the top developed much faster than the other buds, and the differentiation of primary and secondary branches occurred from the top to the bottom (Table 1). When the mother stem was at the yellow ripe stage, most of the axillary buds at the top 2nd and 3rd nodes entered into secondary branch differentiation, while the axillary buds at the top 4th and 5th entered into primary branch differentiation (Fig. 1, Table 1). The observation on the morphology of vegetative organs from regenerated tillers of Shanyou 63 also suggested the superior growth of upper buds in ratoon rice (Additional file 1: Figure S1, Tables 2, 3, 4, 5), which is regulated by hormones.

BRs play important role in plant growth and development including shoot growth, root development, fertility, seed germination and so on. And they also regulate cell division, elongation and differentiation at cellular level [22, 26, 27]. By establishing a comprehensive proteome map of the rice axillary bud before and after the yellow ripe stage, we identified some proteins involved in steroid biosynthesis that were significantly increased. Among them, four took part in BR biosynthesis (Additional file 1: Figure S4). Thus, BR signaling may play a role in the germination of axillary buds in ratoon rice. Thus, our novel data sheds light on the molecular mechanisms underlying BR signaling and may allow researchers to explore further the biological functions of endogenous BRs in the germination of axillary buds in ratoon rice.

## Conclusions

Ratoon rice is a kind of one-season rice cultivated by using axillary buds on rice stakes to germinate and panicle after harvesting in the first season. It has the advantages of planting once, harvesting twice and saving labor and cost. However, compared to the first, few studies on the ratoon rice have been conducted to explore factors effect on yield. As ratoon rice is germinated from axillary buds on rice stakes after harvesting in the first season, so here we investigated the germination mechanism of axillary bud. The axillary buds began to differentiate from bottom to top before heading of the mother stem and developed very slowly. After heading, they differentiated from top to bottom, and the ones on the top developed much faster than others, especially the top 2nd node, of which the average length and dry weight of axillary buds significantly higher than other nodes at the yellow ripe stage. They differentiated into pistil and stamen first at 6 days after yellow ripe stage. Through establishing a comprehensive proteome map of the rice axillary buds at the top 2nd node before and after yellow ripe stage, we found some proteins took part in steroid biosynthesis were significantly increased. Among them, 4 took part in brassinosteroid biosynthesis. Thus, BRs signalling may play a role on the germination of axillary buds in ratoon rice. Together, our novel data shed light on the molecular mechanisms underlying BRs signalling, and may allow researchers to explore further the biological functions of endogenous BRs in the germination of axillary buds in ratoon rice.

## Methods

### Plant materials and growth conditions

Plant materials including Shanyou 63, Zhenshan97, Minghui63 used in this study were preserved in Rice Research Institute, Fujian Academy of Agricultural Sciences. Shanyou 63 is an elite mega rice hybrid grown in China [28]. It is a late-maturing hybrid rice variety with a strong ratooning ability and is cultivated for ratoon rice. It is the product of a cross between the sterile line Zhenshan 97A (weak ratooning ability) and the *indica* restorer line Minghui 63 (strong ratooning ability) [29]. The three varieties were grown in a paddy field in Sanming City, Youxi County, Fujian Province, China and used throughout the experiments. Field management was carried out in accordance with local standard methods. The first season was harvested in August, and the ratooning season was harvested in October. Axillary buds at the top 2nd and 3rd nodes of Minghui 63, Zhenshan 97B and Shanyou 63 with three replicates were harvested and stored at − 20 °C for proteomics analyses.

### Development dynamics of axillary buds

In the first season, 10 tillers of Shanyou 63 were excavated every week from 10 d after the jointing stage to the heading stage, and the differentiation process of axillary buds at different nodes was studied by optical microscopic observation. At 12, 9, 6 and 3 d before the yellow ripe stage, during the yellow ripe stage, and 3 and 6 d after the yellow ripe stage, 30 tillers were excavated per time. The axillary buds at different nodes were decomposed, and the differentiation process of young panicles was examined by microscopy using 10 axillary buds at different nodes. The lengths and dry weights of 20 axillary buds at different nodes were measured with three replicates.

We focused on observing and comparing the differentiation and growth dynamics of axillary buds at different nodes, and we determined trends related to their growth. By summarizing these trends, the sprouting mechanisms of axillary buds could be studied, and then, the mechanisms promoting the high yield of ratoon rice could be clarified. To determine the sprouting mechanisms of axillary buds, we identified genes related to axillary bud sprouting and then explored their specific regulatory mechanisms. Finally, our results provide a basis for the development of technology to positively regulate axillary bud growth.

### Observation of pollen grain morphology

From the flag leaf extension to the full panicle stage during the ratooning season, five tillering panicles of Shanyou 63 were taken from different nodes every 3 d, fixed in FAA solution for 24 h, and then stored in 70% alcohol. Before microscopic examination, the rice panicles were dipped in 50% alcohol for 30 min, 30% alcohol for 30 min, 10% alcohol for 30 min, and then distilled water. The anthers of 1–2 spikelets in the upper, middle, and lower parts of the panicles were placed on glass slides. The anthers at the pollen mother cell and meiosis stages, and at the initial stage of pollen filling, were stained with acetic acid magenta, and anthers at the initial stage of pollen maturity were stained with I_2_-KI. Anthers were crushed to release the pollen grains and left standing for 10 min. They were then placed under cover glass and photographed under a microscope.

### Protein extraction

Axillary buds at the top 2nd and 3rd nodes of Minghui 63, Zhenshan 97B and Shanyou 63 were ground in liquid nitrogen to powder and then transferred to a 5-mL centrifuge tube. Total protein was extracted according to Saravanan and Rose, with some modifications [30]. Four volumes of lysis buffer (8 mol/L urea, 1% Triton-100, 10 m mol/L dithiothreitol, and 1% Protease Inhibitor Cocktail) was added to the cell powder, followed by sonicating three times on ice using a high intensity ultrasonic processor (Scientz). The remaining debris was removed by centrifugation at 20,000 g at 4 °C for 10 min. After precipitation treatment with 20% trichloroacetic acid, the protein was obtained and washed three times with cold acetone. Finally, the protein dissolved in 8 mol/L urea and was determined using a bicinchoninic acid (BCA) protein assay kit following the manufacturer’s instructions.

### Trypsin digestion

The reduction of the protein solution was performed with dithiothreitol (5 mmol/L) for 30 min at 56 °C. At room temperature in darkness, the sample was alkylated with iodoacetamide (11 mmol/L). Then the mixture of triethylamonium bicarbonat (TEAB, 100 mM) and urea (less than 2 mol/L) was using to diluted the protein. The sample was digested overnight with trypsin at 1:50 (trypsin: protein) mass ratio, and digested for 4 h at 1:100 mass ratio.

### TMT labeling

The peptides were digested by trypsin and desalted using SPE column. After vacuum drying, the peptides were reconstituted in TEAB. Then they were processed following the manufacturer’s instructions of TMT kit (Thermo).

### HPLC fractionation

The tryptic peptides were fractionated using high pH reverse-phase HPLC with an Agilent 300 Extend C18 column (5-μm particles, 4.6-mm i.d., 250-mm length). Briefly, peptides were first separated into 60 fractions using a gradient of 8 to 32% acetonitrile (pH 9.0) over 60 min. Then, the peptides were combined into nine fractions and dried by vacuum centrifuging.

### LC- tandem mass spectrometry (MS/MS) analysis

Tryptic peptides were dissolved in solvent A containing 0.1% formic acid. Then, the samples were loaded onto analytical reverse-phase column (15-cm length, 75-μm i.d.). The mobile phase was solvent B (0.1% formic acid in 98% acetonitrile) in gradient elution, and the flow rate was 400 nL/min on an EASY-nLC 1000 UPLC system. Then it was performed using ultra-performance liquid chromatography (UPLC) coupled to triple-quadrupole tandem mass spectrometry (MS-MS). An electrospray voltage of 2.0 kV was applied. The *m/z* scan range was 350 to 1800 and the intact peptides were detected at a resolution of 70,000. Then peptides were selected for MS/MS by NCE 28, and the fragments were detected at a resolution of 17,500. Using one MS scan and 20 MS/MS scans alternately, a data-dependent procedure was performed with dynamic exclusion time of 15 s. Automatic gain control (AGC) was set at 5E4. Fixed first mass was set as 100 m/z.

### Database search

The data of MS/MS result were analyzed by the Maxquant search engine (v.1.5.2.8). The UniProt database (https://www.uniprot.org/) concatenated with a reverse decoy database were applied to search the tandem mass spectra. Trypsin (Parenzyme) was designated as the cleavage enzyme, which was permitted two missing cleavages. In the first search and main search, the mass tolerance for precursor ions was set as 20 ppm and 5 ppm respectively. Meanwhile, the mass tolerance for fragment ions was set as 0.02 Da. Carbamidomethyl on cysteine (Cys) was considered to be a fixed modification, and oxidation on methionine (Met) was identified as a variable modification. The minimum score for a peptide was set > 40, and the false discovery rate (FDR) was adjusted to < 1%.

### Bioinformatics methods

GO is a major bioinformatics initiative to unify the representation of genes and gene product attributes across all species [31]. The Go annotation was performed using UniProt-GOA database. After converted to UniProt IDs, the protein IDs were mapped to GO IDs. Wolfpsort, an updated version of PSORT/PSORT II for the prediction of eukaryotic sequences, was used as a subcellular localization predication software. The KEGG database was used to annotate protein pathways [32]. First, the KEGG online service tool KAAS was used to annotate protein KEGG database descriptions. Then, the annotation result was mapped on the KEGG pathway database using the KEGG online service tool KEGG mapper.

A functional enrichment pathway analysis, including the enrichment of the GO analysis, and a protein domain analysis were used to determine whether differentially abundant proteins had significant enrichment trends in particular functional types. The proteins were classified into three categories including biological process, cellular compartment and molecular function by GO annotation. The enrichment levels of the differentially expressed proteins against all the identified proteins were determined by a two-tailed Fisher’s exact test, and a GO term with a corrected *p*-value < 0.05 was considered as significant term. Enrich pathways were identified using KEGG database. The enrichment levels were determined by a two-tailed Fisher’s exact test and a pathway with a corrected p-value < 0.05 was considered as significant pathway. Enrichment analysis of protein domain was performed using InterPro database (https://www.ebi.ac.uk/interpro/). The enrichment levels were determined by a two-tailed Fisher’s exact test and protein domains with *p*-values < 0.05 were considered as significantly enriched.

### RNA isolation and qRT-PCR analysis

Total RNA was isolated from axillary buds of Minghui 63, Zhenshan 97B, and Shanyou 63 using TRI-zol reagent [33]. First-strand cDNA was synthesized from 2 μg of total RNA using a high capacity cDNA reverse transcription kit (ABI, Foster City, CA, USA) following the manufacturer’s instructions. The nucleotide sequences of genes that we concerned were downloaded from http://plants.ensembl.org/info/website/ftp/index.html. Primers used for qRT-PCR analysis were designed, subsequently tested in a dissociation curve analysis and verified for the absence of nonspecific amplification. Primers are listed in Additional file 1: Table S1. The qRT-PCR analysis was performed using the Thermo Lifetech ABI QuantStudio 6 Flex sequence detection system and a SYBR Green real-time PCR mix (Roche, Shanghai, China). The relative quantification of gene expression was performed using *actin* gene expression as a reference. Data were analyzed using Thermo Lifetech ABI QuantStudio 6 Flex SDS software.

## Supplementary information

**Additional file 1 : Figure S1.** The shape of axillary buds at different nodes of the hybrid rice cultivar Shanyou 63. (A) The yellow ripe stage of the first crop. (B) 3 d after the yellow ripe stage of the first crop. **Figure S2.** Pie charts of subcellular classifications of the differentially abundant proteins. Different colors represent different subcellular localizations. **Figure S3.** Protein domain enrichment analysis. *p* < 0.05. **Figure S4.** Steroid biosynthetic pathway in rice axillary buds at 3 d after the yellow ripe stage. Proteomic data were used to construct the steroid biosynthetic pathway. The numbers represent the protein IDs, and increased proteins are indicated in red. Broken arrows indicate multiple steps between two compounds. **Table S1**. List of primers used in qRT-PCR analysis. **Table S2.** Differentially expressed proteins of rice axillary buds at the top 2nd node before and after yellow ripe stage.

## Data Availability

Not applicable.

## Abbreviations

1-DE: One-dimensional electrophoresis
BR: Brassinosteroid
BRs: Brassinosteroids
GO: Gene ontology
KEGG: The Kyoto Encyclopedia of Genes and Genomes database
qRT-PCR: Real-time quantitative reverse transcription PCR
TEAB: Triethylamonium bicarbonat
TMT: Tandem Mass Tag
