# Supplementary material for: Brassinosteroid signaling may regulate the germination of axillary buds in ratoon rice
Source: BMC Plant Biol. 2020 Feb 14;20:76. doi: 10.1186/s12870-020-2277-x (PMC7023735; doi:10.1186/s12870-020-2277-x)
Supplement: Supplementary file 1 — Additional file 1 : Figure S1. The shape of axillary buds at different nodes of the hybrid rice cultivar Shanyou 63. (A) The yellow ripe stage of the first crop. (B) 3 d after the yellow ripe stage of the first crop. Figure S2. Pie charts of subcellular classifications of the differentially abundant proteins. Different colors represent different subcellular localizations. Figure S3. Protein domain enrichment analysis. p < 0.05. Figure S4. Steroid biosynthetic pathway in rice axillary buds at 3 d after the yellow ripe stage. Proteomic data were used to construct the steroid biosynthetic pathway. The numbers represent the protein IDs, and increased proteins are indicated in red. Broken arrows indicate multiple steps between two compounds. Table S1. List of primers used in qRT-PCR analysis. Table S2. Differentially expressed proteins of rice axillary buds at the top 2nd node before and after yellow ripe stage. [file 12870_2020_2277_MOESM1_ESM.docx]

Supplementary materials


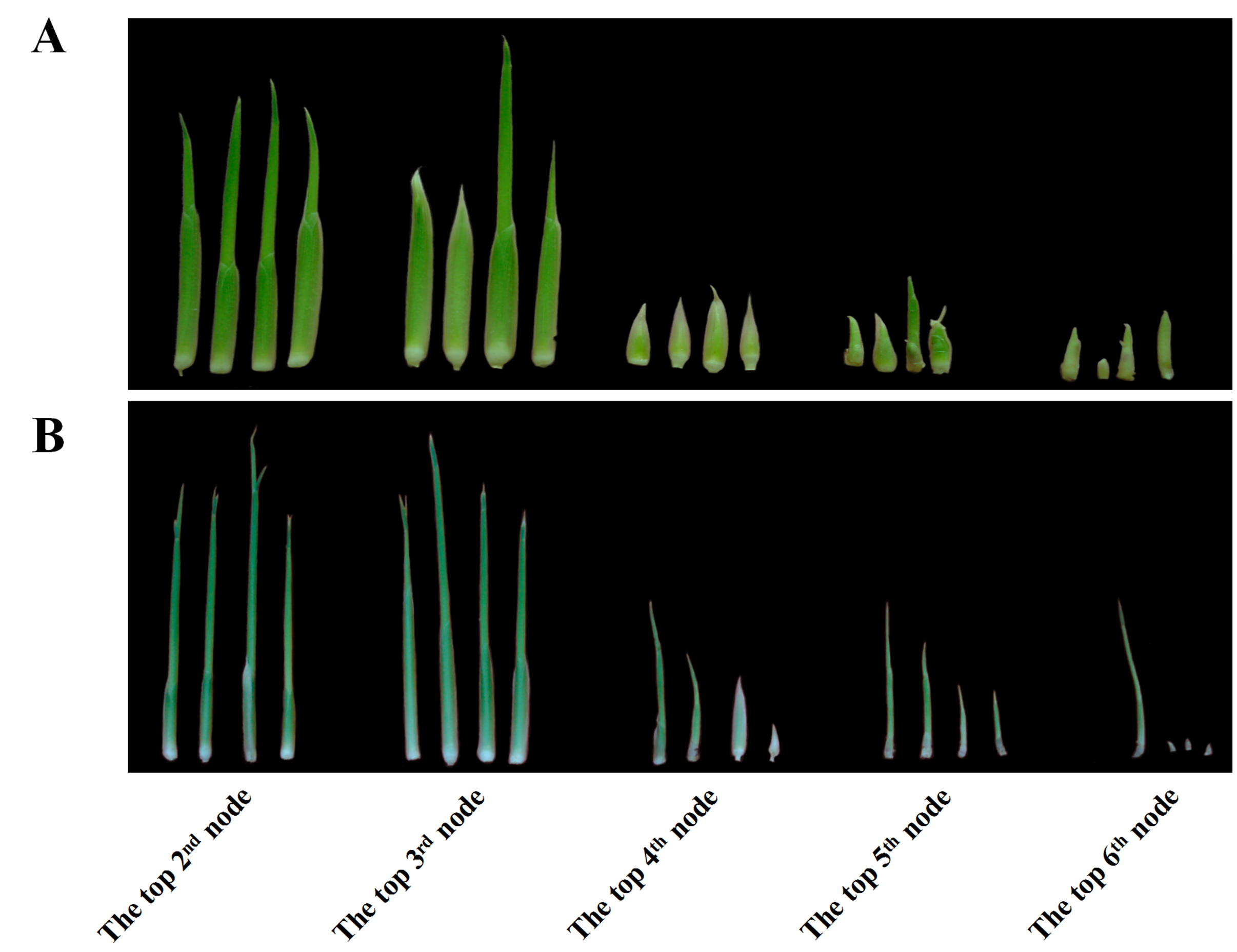


Fig. S1. The microscopic observations of the differentiation processes of axillary buds. (A) Axillary buds during the bract differentiation stage. (B) Bract differentiation stage (The prophyll is 6-10 mm long). (C) Bract differentiation stage (The first leaf is 4-7 mm long). (D) Bract differentiation stage (The second leaf is 1-2 mm long). (E) Bract differentiation stage (the third leaf is 0.2-0.5 mm long). (F) Bract differentiation stage (The first bract is 0.1-0.2 mm long). (G) Primary branch differentiation (0.1-0.5 mm, the prophyll is 20-30 mm long). (H) Secondary branch differentiation (0.5-1.2 mm, the prophyll is 40-60 mm long). (I) Spikelet differentiation (1.0-2.5 mm). (J) Closeup of spikelet differentiation. (K) The initial stage of pistil and stamen differentiation (3-5 mm). (L) The late stage of pistil and stamen differentiation (10 mm).


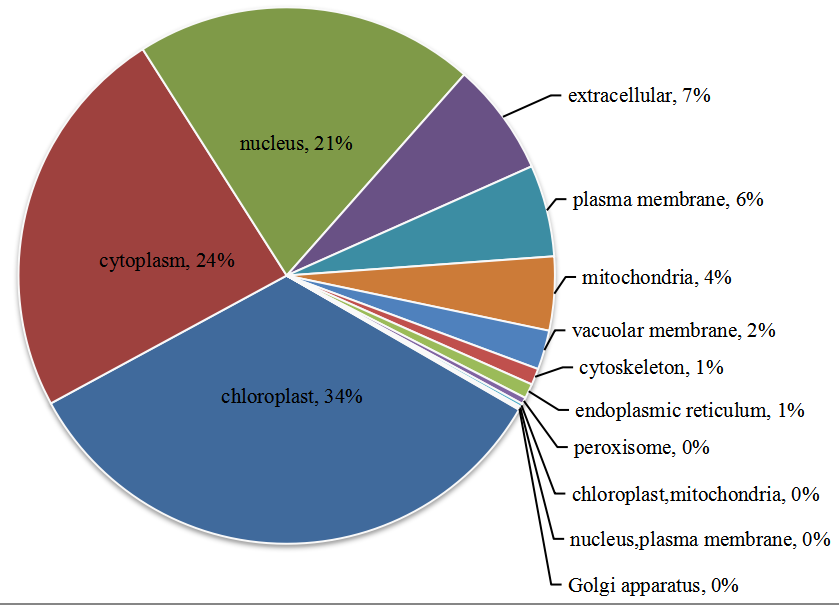


Fig. S2. Pie charts of subcellular classifications of the differentially abundant proteins. Different colors represent different subcellular localizations.


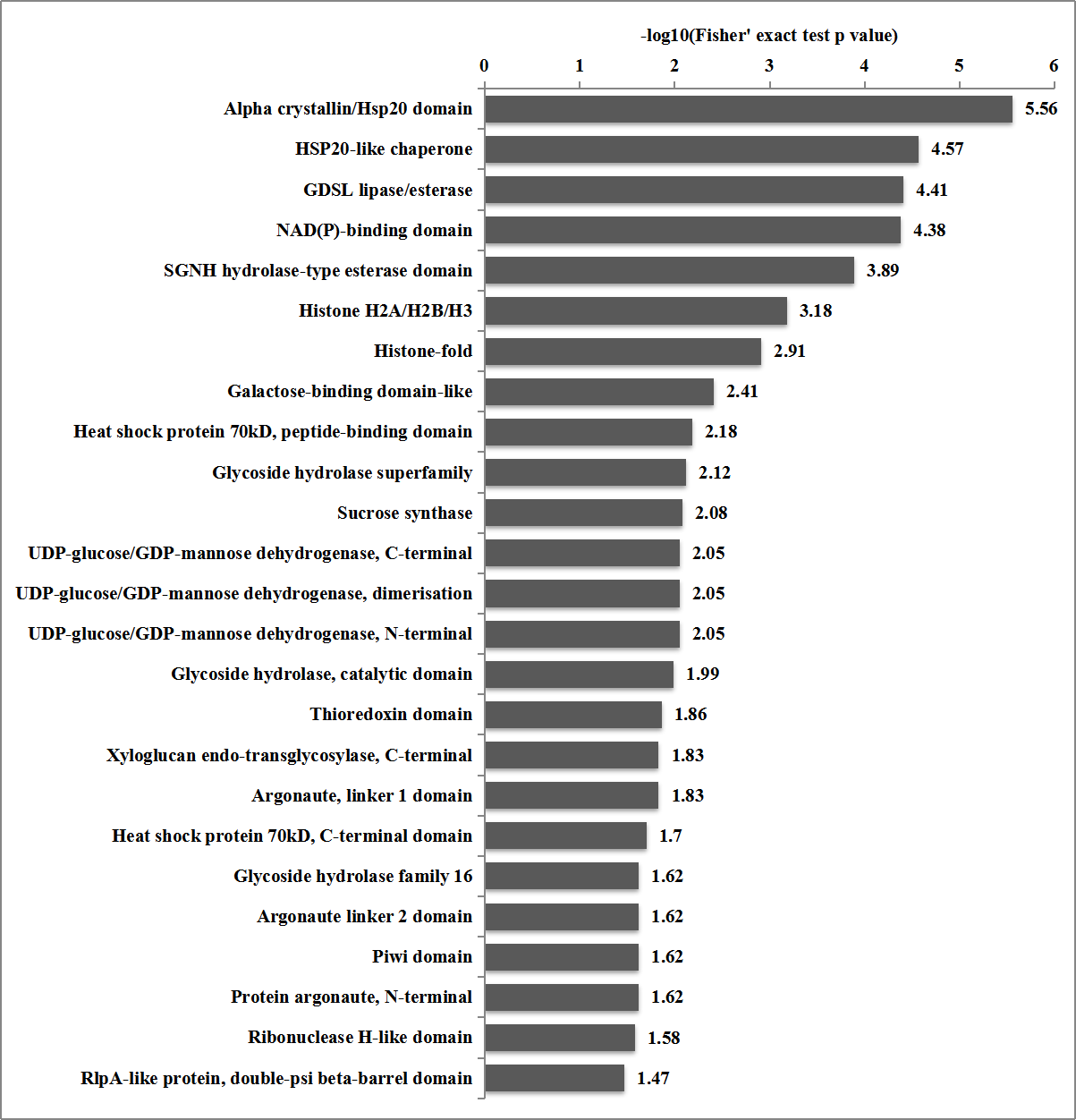


Fig. S3. Protein domain enrichment analysis. *p* < 0.05.


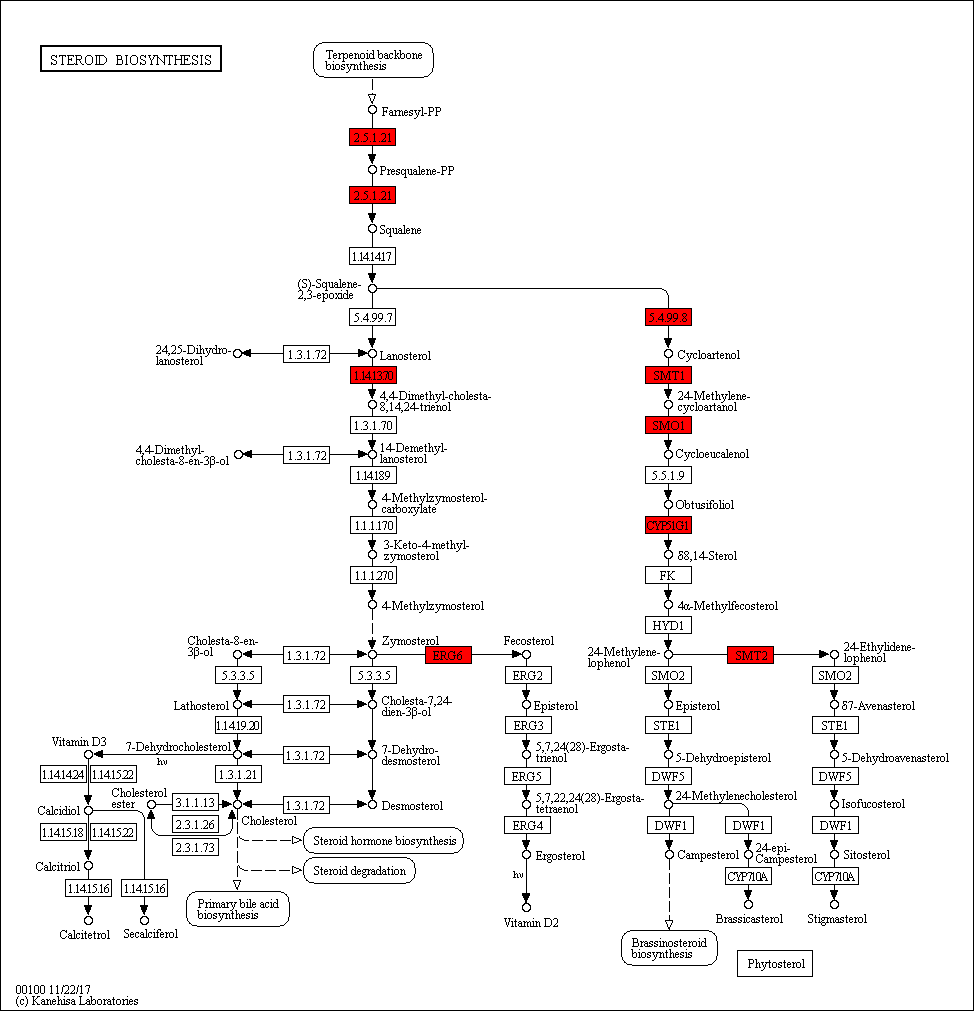


Fig. S4. Steroid biosynthetic pathway in rice axillary buds at 3 d after the yellow ripe stage. Proteomic data were used to construct the steroid biosynthetic pathway. The numbers represent the protein IDs, and increased proteins are indicated in red. Broken arrows indicate multiple steps between two compounds.

**Table S1. List of primers used in qRT-PCR analysis**

| **Purpose Gene** | **Sense primer (5'→3')** | **Anti-sense primer (5'→3')** |
| --- | --- | --- |
| BGIOSGA007157 | CGGATGGTTTATTTGCCTATGTGTTAC | CGAGCCTTGTCCCAATCAACTTCAT |
| BGIOSGA035444 | GAGTCCTTCCTTGTTGCTGTGGTT | GTACATTCTTGCCTTCGGATCGTT |
| BGIOSGA011034 | CAGGAGACAGAGCCACTCGGAA | ATGGGACTTGTGGAACTGGATGAG |
| BGIOSGA017490 | CCAATAAGGAGAGGTGGTGAGGGC | AGCATCTGAGGTGAGCACACATTG |
| BGIOSGA026375 | GCTGCTACGACGAGGGGAAGAGG | CTGGGCGACAAAGTTGCTGACGA |
| BGIOSGA008810 | ATCTGTCCAATCCTCGCAACTTCA | GTGAGGTTTCTCTTTGCCATTTCG |
| BGIOSGA017637 | CAATCGTGGTCTCTCTGTGGTCG | ACCCGAAACCAACAAGGCTGACC |
| BGIOSGA006239 | TACCCTGAGCACCCTCTTTTACCT | CCCCTGTACCAACCTTCTTCTCAA |
| Actin150 | AGTGTCTGGATTGGAGGAT | TCTTGGCTTAGCATTCTTG |

| **Compare group** | **Regulated type** | **fold change >1.2** | **fold change >1.3** | **fold change >1.5** | **fold change >2** |
| --- | --- | --- | --- | --- | --- |
| **Aa1/Ba1** | **up-regulated** | **422** | **203** | **74** | **17** |
|  | **down-regulated** | **581** | **407** | **225** | **87** |
| **Aa2/Aa1** | **up-regulated** | **169** | **75** | **18** | **2** |
|  | **down-regulated** | **130** | **49** | **21** | **5** |
| **Aa2/Ba2** | **up-regulated** | **185** | **92** | **37** | **6** |
|  | **down-regulated** | **269** | **140** | **59** | **21** |
| **Aa3/Aa1** | **up-regulated** | **745** | **525** | **318** | **117** |
|  | **down-regulated** | **462** | **246** | **85** | **19** |
| **Aa3/Aa2** | **up-regulated** | **847** | **633** | **383** | **156** |
|  | **down-regulated** | **623** | **394** | **137** | **27** |
| **Aa3/Ba3** | **up-regulated** | **713** | **501** | **260** | **88** |
|  | **down-regulated** | **661** | **422** | **218** | **83** |
| **Ba2/Ba1** | **up-regulated** | **230** | **94** | **24** | **3** |
|  | **down-regulated** | **371** | **216** | **98** | **23** |
| **Ba3/Ba1** | **up-regulated** | **380** | **243** | **136** | **45** |
|  | **down-regulated** | **338** | **239** | **121** | **35** |
| **Ba3/Ba2** | **up-regulated** | **489** | **323** | **146** | **52** |
|  | **down-regulated** | **295** | **146** | **47** | **15** |
| **Ca1/Ba1** | **up-regulated** | **260** | **169** | **93** | **35** |
|  | **down-regulated** | **331** | **200** | **90** | **31** |
| **Ca2/Ba2** | **up-regulated** | **616** | **458** | **250** | **102** |
|  | **down-regulated** | **317** | **142** | **47** | **11** |
| **Ca2/Ca1** | **up-regulated** | **372** | **269** | **167** | **61** |
|  | **down-regulated** | **147** | **60** | **14** |  |
| **Ca3/Ba3** | **up-regulated** | **727** | **495** | **254** | **65** |
|  | **down-regulated** | **767** | **518** | **226** | **39** |
| **Ca3/Ca1** | **up-regulated** | **857** | **619** | **360** | **124** |
|  | **down-regulated** | **836** | **576** | **264** | **56** |
| **Ca3/Ca2** | **up-regulated** | **761** | **569** | **343** | **123** |
|  | **down-regulated** | **1031** | **822** | **480** | **137** |

**Table S2. Differentially expressed proteins of rice axillary buds at the top 2^nd^ node before and after** **yellow ripe stage**
